# Efficacy and Safety of Avapritinib in Treating Unresectable or Metastatic Gastrointestinal Stromal Tumors: A Phase I/II, Open-Label, Multicenter Study

**DOI:** 10.1093/oncolo/oyac242

**Published:** 2022-12-07

**Authors:** Jian Li, Xinhua Zhang, Yanhong Deng, Xin Wu, Zhichao Zheng, Yongjian Zhou, Shirong Cai, Yanqiao Zhang, Jun Zhang, Kaixiong Tao, Yuehong Cui, Hui Cao, Kuntang Shen, Jiren Yu, Ye Zhou, Wenxiao Ren, Chenglin Qu, Wanqi Zhao, Jin Hu, Wei Wang, Jason Yang, Lin Shen

**Affiliations:** Department of Gastrointestinal Oncology, Laboratory of Carcinogenesis and Translational Research of the Ministry of Education, Peking University School of Oncology, Beijing Cancer Hospital & Institute, Beijing, People’s Republic of China; Department of Gastrointestinal Surgery, The First Affiliated Hospital, Sun Yat-sen University, Guangzhou, People’s Republic of China; Department of Medical Oncology, The Sixth Affiliated Hospital, Sun Yat-sen University, Guangzhou, People’s Republic of China; Department of General Surgery, Chinese PLA General Hospital, Beijing, People’s Republic of China; Department of Gastrosurgery, Liaoning Cancer Hospital & Institute, Shenyang, People’s Republic of China; Department of Gastroenterology, Fujian Medical University Union Hospital, Fuzhou, People’s Republic of China; Department of Gastrointestinal Surgery, The First Affiliated Hospital, Sun Yat-sen University, Guangzhou, People’s Republic of China; Department of Gastrointestinal Medical Oncology, Harbin Medical University Cancer Hospital, Harbin, People’s Republic of China; Department of Gastroenterology, The First Affiliated Hospital of Chongqing Medical University, Chongqing, People’s Republic of China; Department of Gastroenterology, Wuhan Union Hospital, Tongji Medical College, Huazhong University of Science and Technology, Wuhan, People’s Republic of China; Department of Medical Oncology, Fudan University Zhongshan Hospital, Shanghai, People’s Republic of China; Department of Gastroenterology, Renji Hospital Affiliated to Shanghai Jiaotong University School of Medicine, Shanghai, People’s Republic of China; Department of General Surgery, Fudan University Zhongshan Hospital, Shanghai, People’s Republic of China; Department of Gastroenterology, The First Affiliated Hospital, Zhejiang University, Hangzhou, People’s Republic of China; Department of Gastric Surgery, Fudan University Shanghai Cancer Center, Shanghai, People’s Republic of China; CStone Pharmaceuticals (Suzhou), Suzhou, People’s Republic of China; CStone Pharmaceuticals (Suzhou), Suzhou, People’s Republic of China; CStone Pharmaceuticals (Suzhou), Suzhou, People’s Republic of China; CStone Pharmaceuticals (Suzhou), Suzhou, People’s Republic of China; CStone Pharmaceuticals (Suzhou), Suzhou, People’s Republic of China; CStone Pharmaceuticals (Suzhou), Suzhou, People’s Republic of China; Department of Gastrointestinal Oncology, Laboratory of Carcinogenesis and Translational Research of the Ministry of Education, Peking University School of Oncology, Beijing Cancer Hospital & Institute, Beijing, People’s Republic of China

**Keywords:** tyrosine kinase inhibitor, avapritinib, gastrointestinal stromal tumors, PDGFRA D842V, fourth-line therapy

## Abstract

**Background:**

Avapritinib is a type 1 kinase inhibitor designed to potently and selectively inhibit oncogenic KIT/PDGFRA mutants by targeting the kinase active conformation. This multicenter, single-arm, open-label, phase I/II bridging study of NAVIGATOR in Chinese patients evaluated the safety and the antineoplastic activity of avapritinib in Chinese patients with unresectable/metastatic gastrointestinal stromal tumors (GIST).

**Methods:**

Phase I comprised dose escalation for safety and phase II dose determination. Phase II comprised dose expansion for safety/efficacy evaluations in patients with PDGFRA D842V mutations or patients having received at least 3 lines of therapy without PDGFRA D842V mutations. The primary endpoints were recommended phase II dose, safety, and Independent Radiology Review Committee (IRRC)-assessed objective response rate (ORR).

**Results:**

No dose-limiting toxicities occurred (*n* = 10); the recommended phase II dose was avapritinib 300 mg once daily orally. Fifty-nine patients initially received avapritinib 300 mg. Common grade ≥3 treatment-related adverse events were anemia, decreased white blood cell count, increased blood bilirubin levels, and decreased neutrophil count. In patients with PDGFRA D842V mutations, IRRC- and investigator-assessed ORRs were 75% and 79%, respectively; clinical benefit rates were both 86%. Median duration of response/progression-free survival were not reached. IRCC- and investigator-assessed ORRs in patients in the fourth- or later-line setting were 22% and 35%, respectively. Median progression-free survivals were 5.6 months for both. Overall survival data were immature and not calculated.

**Conclusion:**

Avapritinib was generally well tolerated and showed marked anti-tumor activity in Chinese patients with GIST bearing PDGFRA D842V mutations and notable efficacy as fourth- or later-line monotherapy (ClinicalTrials.gov Identifier: NCT04254939).

## Teaser

This article evaluates the safety and the antineoplastic activity of avapritinib in Chinese patients with unresectable/metastatic gastrointestinal stromal tumors.

**Lessons Learned**

- This open-label, phase I/II bridging study indicated that avapritinib was safe and tolerable for patients with unresectable or metastatic gastrointestinal stromal tumors (GIST).
- Avapritinib provided pronounced anti-tumor activity in patients with PDGFRA D842V mutations and in patients without such mutations but receiving fourth- or later-line treatment.
- Avapritinib was approved in China for the treatment of PDGFRA D842V-mutant GIST based on these and the global NAVIGATOR findings.

## Discussion

Avapritinib (BLU-285) is a potent and selective small-molecule inhibitor that targets KIT proto-oncogene receptor tyrosine kinase (KIT) exon 17 and platelet-derived growth factor receptor alpha (PDGFRA) exon 18 mutations via type 1 inhibition.^1-3^ Preclinical studies demonstrated robust in vitro and in vivo activity of avapritinib against clinically relevant KIT primary and resistant mutants.^4^ Avapritinib was first approved in 2020 in the US for unresectable or metastatic gastrointestinal stromal tumors (GIST) with PDGFRA exon 18 mutations.

The safety and preliminary clinical efficacy of avapritinib for PDGFRA D842V-mutant GISTs were demonstrated in the global, phase I Avapritinib in Advanced PDGFRA D842V-mutant Gastrointestinal Stromal Tumour (NAVIGATOR) study.^5^ An individual, open-label, phase I/II bridging study was conducted in China aiming to determine the RP2D, to evaluate the safety, and to assess the efficacy of avapritinib in Chinese patients with unresectable or metastatic GIST. Here we report the efficacy of avapritinib in patients with PDGFRA D842V mutations regardless of prior lines of treatment, and in patients without PDGFRA D842V mutations receiving fourth- or later-line treatment.

At data cutoff (January 8, 2021), 65 patients had received at least one avapritinib dose (12 patients in phase I, 53 in phase II), with 59 patients initially receiving avapritinib 300 mg (Table 1).

**Table 1.** Patient characteristics.

Characteristic	Avapritinib starting dose		
	200 mg *n* = 6	300 mg *n* = 59	Total *N* = 65
Age (years)			
Mean (standard deviation)	58 (11)	59 (9)	59 (9)
Median (min, max)	60 (45, 68)	62 (43, 74)	62 (43, 74)
<65, *n* (%)	3 (50)	40 (68)	43 (66)
≥65, *n* (%)	3 (50)	19 (32)	22 (34)
Sex, *n* (%)			
Male	6 (100)	38 (64)	44 (68)
Female	0	21 (36)	21 (32)
ECOG performance status, *n* (%)			
0	1 (17)	16 (27)	17 (26)
1	5 (83)	43 (73)	48 (74)
Largest target lesion size at baseline, *n* (%)			
≤5 cm	4 (67)	23 (39)	27 (42)
5–10 cm	1 (17)	25 (42)	26 (40)
>10 cm	1 (17)	10 (17)	11 (17)
Missing	0	1 (2)	1 (2)
Primary tumor location, *n* (%)			
Stomach	0	24 (41)	24 (37)
Rectum	0	2 (3)	2 (3)
Omentum	0	2 (3)	2 (3)
Small intestine	6 (100)	23 (39)	29 (45)
Peritoneum	0	2 (3)	2 (3)
Other	0	6 (10)	6 (9)
Histology/cytology type, *n* (%)			
Spindle cell	2 (33)	37 (63)	39 (60)
Epithelioid	0	6 (10)	6 (9)
Mixed spindled-epithelioid	1 (17)	7 (12)	8 (12)
Missing	3 (50)	9 (15)	12 (19)
Cancer stage at screening (TNM), *n* (%)			
Stage IV	6 (100)	57 (97)	63 (97)
Other	0	2 (3)	2 (3)
Number of previous TKIs, *n* (%)			
0	0	7 (12)	7 (11)
1	0	12 (20)	12 (19)
2	1 (17)	16 (27)	17 (26)
3	4 (67)	18 (31)	22 (34)
4 or more	1 (17)	6 (10)	7 (11)
Metastases diagnosed, *n* (%)			
Yes	6 (100)	58 (98)	64 (99)
No	0	1 (2)	1 (2)
Time since initial diagnosis (years)^a^			
Mean (standard deviation)	6 (3)	6 (4)	6 (4)
Median (min, max)	6 (2, 10)	5 (0, 16)	5 (0, 16)

a Time since initial diagnosis calculated by: (date of the first administration of treatment − date of the initial diagnosis + 1 day)/365.25 days.

Abbreviations: ECOG, Eastern Cooperative Oncology Group; TKI, tyrosine kinase inhibitor; TNM, tumor-node-metastasis.

Avapritinib had manageable safety and tolerability, consistent with NAVIGATOR. It demonstrated clinical benefit in Chinese patients with GIST bearing the PDGFRA D842V mutation and promising preliminary anti-tumor activity as fourth- or later-line monotherapy. Avapritinib demonstrated high anti-tumor activity (objective response rate [ORR] 79%) in patients with PDGFRA D842V-mutant GIST, suggesting that it may be effective in first-, or subsequent, lines of treatment in Chinese patients with advanced/metastatic PDGFRA D842V-mutant GIST, which represents a previously untreatable GIST subtype.

**Table:** 

Trial Information	
Disease	Gastrointestinal stromal tumors
Stage of disease/treatment	Unresectable or metastatic
Prior therapy	Patients with GIST bearing *PDGFRA* D842V mutations in any line setting. Patients without *PDGFRA* D842V mutations who have received at least 3 lines of treatment
Type of study	Multicenter, single-arm, open-label
Primary endpoint	The primary endpoints of phase I were recommended phase II dose (RP2D), incidence of dose-limiting toxicities (DLTs), and safety. Adverse events (AEs) of special interest (AESIs) included those inducing cognitive effects or intracranial bleeding. The primary endpoint of phase II was Independent Radiology Review Committee (IRRC)-assessed ORR per mRECIST v1.1
Secondary endpoints	Secondary endpoints included IRRC-assessed ORR (phase I), safety (phase II), and investigator-assessed ORR (phase I/II). Response during phase II was also assessed based on the duration of response (DOR), progression-free survival (PFS), clinical benefit rate (CBR), and 12-month overall survival (OS) rate (phase I/II). Exploratory endpoints included IRRC-assessed ORR per Choi criteria^6^ and OS
Investigator’s analysis	Active and should be pursued further

## Additional Details of Endpoints or Study Design

Phase I used a modified 3 + 3 dose-escalation design for safety and phase II dose determination.

Phase II was a dose-expansion phase for safety and efficacy evaluation. Screening, follow-up, and procedure details are described in the Appendix. This study was initiated in August 2019 and registered with ClinicalTrials.gov, identifier: NCT04254939.

Eligible Chinese patients were ≥18 years of age and had confirmed unresectable or metastatic GIST, at least one measurable lesion defined by the modified Response Evaluation Criteria in Solid Tumors (mRECIST v1.1) for patients with GIST, and an Eastern Cooperative Oncology Group performance status of 0-1. All patients provided written informed consent before study participation.

In phase I, avapritinib was administered orally at a starting dose of 200 mg once daily (QD) for one or more cycles, each consisting of 28 consecutive days. After at least 3 evaluable patients in the first cohort completed the first cycle, DLTs were evaluated; dose escalation to 300 mg QD was based on the available safety data.

For phase I/II, enrollment criteria were unresectable or metastatic GIST confirmed by histology or cytology; either progression after treatment with imatinib and at least one other tyrosine kinase inhibitor (TKI), intolerance, or lack of available standard of care (SOC); and a tumor harboring a mutation in the PDGFRA gene that results in a D842V substitution at the protein level.

The phase II study included 2 cohorts: cohort 1 included patients with unresectable GIST harboring a PDGFRA D842V mutation; cohort 2 included patients with unresectable GIST having received at least 3 lines of therapy and without a PDGFRA D842V mutation.

## Statistical Analysis

Details of sample size calculations and analysis sets are included in the Appendix. Data were described descriptively using frequency and percentage for categorical variables and mean, standard deviation, median, minimum, and maximum for continuous variables. Efficacy analyses were performed separately for the PDGFRA D842V population and patients without PDGFRA D842Vmutations receiving avapritinib as fourth- or later-line treatment.

**Table:** 

Drug Information	
**Generic/working name**	Avapritinib
**Company name**	BLU-285
**Drug type**	Small molecule
Drug class	Tyrosine kinase inhibitor
Dose	200 or 300 mg (phase I) 300 mg (phase II)
**Route**	Oral
Schedule of administration	Once daily

**Table:** 

Patient Characteristics		
	Dose Group Avapritinib 200 mg	Dose Group Avapritinib 300 mg*
Number of patients, male	6	38
Number of patients, female	0	21
Stage	Stage IV: *n* = 6	Stage IV: *n* = 57 Other: *n* = 2
Age: median (range)	60 years (45, 68)	62 years (43, 74)
Number of prior TKIs	0: 0	0: 7
	1: 0	1: 12
	2: 1	2: 16
	3: 4	3: 18
	≥4: 1	≥4: 6
Performance status: ECOG	0: 1	0: 16
	1: 5	1: 43
Cancer Types or Histologic Subtypes	Number	Number
Spindle cell	2	37
Epithelioid	0	6
Mixed spindled-epithelioid	1	7
Missing	3	9

*There were 6 patients from phase I and 53 patients from phase II in this dose group.

**Table:** 

Primary Assessment Method	
Title	Safety
Number of patients screened	73
Number of patients enrolled	65
Number of patients evaluable for toxicity	65

## Outcome Notes: Safety Population

In the safety population, 6 and 59 patients received avapritinib at a starting dose of 200 and 300 mg, respectively (Table 1). The median age (62 years) was similar between groups. All patients (6/6) who received 200 mg avapritinib were male, while in the 300-mg dose group, 38/59 (64%) were male. Overall, the primary tumor locations were the small intestine in 29/65 (45%) patients and the stomach in 24/65 (37%) patients, and most tumors were of the spindle cell type (39/65; 60%).

### DLT and RP2D

 Among the 12 patients in the phase I study, 10 met the requirements for inclusion in the dose-determining set, including 6 patients in the 200-mg dose group and four patients in the 300-mg dose group. All 10 patients completed the DLT assessment and no DLT events were observed. The RP2D was determined to be 300 mg QD orally.

### Phase I/II Safety

 The median treatment duration of avapritinib in both dose groups was 36.0 weeks (range = 2.0-72.0 weeks). Among the 65 patients, 30 (50.8%), all from the 300-mg group, had a dose reduction; and 56 (86.2%) patients had a dose interruption. TEAEs leading to study drug discontinuation occurred in 3/65 (5%) patients (Table 3) and none were related to avapritinib. All patients in both dose groups experienced at least one treatment-related adverse event (TRAE) of any grade (Table 2). The most commonly reported TRAE of grade ≥3, in both dose groups, was anemia (23/65; 35%). The most commonly reported TRAEs of grade ≥3 in the 300-mg dose group were decreased white blood cell count (11/59; 19%), increased blood bilirubin levels (8/59; 14%), and decreased neutrophil count (7/59; 12%). TRAEs occurred in 6/6 (100%) patients in the 200-mg dose group and 58/59 (98%) patients in the 300-mg dose group (Table 3). Grade ≥3 TRAEs were reported in 47/65 (72%) patients in total. Grade 4 TRAEs were observed in 2 patients from the 300-mg dose group and no grade 5 TRAEs were reported.

**Table 2.** Summary of treatment-related adverse events

	Avapritinib starting dose					
	200 mg *n* = 6		300 mg *n* = 59		Total *N* = 65	
	Grade ≥3	All grades^a^	Grade ≥3	All grades^a^	Grade ≥3	All grades^a^
Any TRAE, *n* (%)	1 (17)	6 (100)	46 (78)	58 (98)	47 (72)	64 (99)
Anemia	1 (17)	4 (67)	22 (37)	48 (81)	23 (35)	52 (80)
Blood bilirubin increased	0	4 (67)	8 (14)	45 (76)	8 (12)	49 (75)
White blood cell count decreased	0	5 (83)	11 (19)	33 (56)	11 (17)	38 (59)
Blood CPK increased	1 (17)	6 (100)	4 (7)	24 (41)	5 (8)	30 (46)
Face edema	0	1 (17)	2 (3)	28 (48)	2 (3)	29 (45)
AST increased	0	4 (67)	0	23 (39)	0	27 (42)
Hair color changes	0	1 (17)	0	26 (44)	0	27 (42)
Eyelid edema	0	6 (100)	1 (2)	20 (34)	1 (2)	26 (40)
Neutrophil count decreased	0	2 (33)	7 (12)	24 (41)	7 (11)	26 (40)
Periorbital edema	0	0	3 (5)	20 (34)	3 (5)	20 (31)
Edema peripheral	0	5 (83)	1 (2)	13 (22)	1 (2)	18 (28)
Lacrimation increased	0	0	0	13 (22)	0	13 (20)
Nausea	0	0	0	13 (22)	0	13 (20)
Memory impairment	0	0	0	12 (20)	0	12 (19)
Rash	0	2 (33)	0	9 (15)	0	11 (17)
ALT increased	0	2 (33)	1 (2)	8 (14)	1 (2)	10 (15)
Diarrhea	0	1 (17)	0	9 (15)	0	10 (15)
Leukopenia	0	0	3 (5)	10 (17)	3 (5)	10 (15)
Neutropenia	0	0	5 (9)	10 (17)	5 (8)	10 (15)
Platelet count decreased	0	2 (33)	1 (2)	8 (14)	1 (2)	10 (15)
Decreased appetite	0	0	0	9 (15)	0	9 (14)
Lymphocyte count decreased	0	0	2 (3)	9 (15)	2 (3)	9 (14)
Blood creatinine increased	0	1 (17)	0	7 (12)	0	8 (12)
Dizziness	0	0	0	8 (14)	0	8 (12)
Fatigue	0	0	1 (2)	8 (14)	1 (2)	8 (12)
Insomnia	0	0	0	8 (14)	0	8 (12)
Malaise	0	0	3 (5)	8 (14)	3 (5)	8 (12)
Alopecia	0	0	0	7 (12)	0	7 (11)
GERD	0	0	0	6 (10)	0	6 (9)
Headache	0	0	0	6 (10)	0	6 (9)
Hypokalemia	0	0	1 (2)	6 (10)	1 (2)	6 (9)
Hypophosphatemia	0	0	0	6 (10)	0	6 (9)
Vomiting	0	0	1 (2)	6 (10)	1 (2)	6 (9)
Bilirubin conjugated increased	0	0	1 (2)	4 (7)	1 (2)	4 (6)
Blood lactate dehydrogenase increased	0	0	4(7)	4 (7)	4(6)	4 (6)
Cognitive disorder	0	0	1(2)	4 (7)	1(2)	4 (6)
Generalized edema	0	0	4(7)	4 (7)	4(6)	4 (6)
Thrombocytopenia	0	0	4(7)	4 (7)	4(6)	4 (6)

^a^Includes all TRAEs reported by ≥5 patients in total.

Abbreviations: ALT, alanine aminotransferase; AST, aspartate aminotransferase; CPK, creatine phosphokinase; GERD, gastroesophageal reflux disease; TRAE, treatment-related adverse event.

**Table 3.** Summary of treatment-emergent adverse events (safety analysis set)

	Avapritinib starting dose		
	200 mg	300 mg	Total
	(*n* = 6)	(*n* = 59)	(*n* = 65)
Any TEAE	6 (100)	59 (100)	65 (100)
TRAE	6 (100)	58 (98)	64 (99)
Grade ≥3 TEAE	5 (83)	52 (88)	57 (88)
Grade ≥3 TRAE	1 (17)	46 (78)	47 (72)
Serious TEAE	4 (67)	28 (48)	32 (49)
Serious TRAE	—	15 (25)	15 (23)
TEAE leading to death	3 (50)	8 (14)	11 (17)
TEAE leading to study drug discontinuation	1 (17)	2 (3)	3 (5)
TEAE leading to dose reduction	—	3 (5)	3 (5)
TEAE leading to drug interruption	4 (67)	48 (81)	52 (80)
AESI – cognitive effects	—	16 (27)	16 (25)
AESI – intracranial bleeding	—	2 (3)	2 (3)

Data are shown as *n* (%).

Abbreviations: AESI, adverse event of special interest; TEAE, treatment-emergent adverse event; TRAE, treatment-related adverse event.

TEAEs were defined as any adverse event that occurred or worsened on or after the initiation of the study drug. For frequency counts by System Organ Class or Preferred Term, multiple occurrences of the same condition in an individual were counted only once.

Serious TEAEs were observed in 4/6 (67%) patients in the 200-mg dose group and 28/59 (48%) patients in the 300-mg dose group. Serious TRAEs were reported in 15/65 (23%) patients in total. The most commonly occurring serious TRAEs at any dose were anemia (7/65; 11%) and cognitive disorder (3/65; 5%) (Table 4).

**Table 4.** Serious treatment-related adverse events and adverse events of special interest (safety analysis set)

	Avapritinib starting dose		
	200 mg *n* = 6	300 mg *n* = 59	Total *N* = 65
Patients with at least one serious TRAE			
Anemia	0	7 (12)	7 (11)
Cognitive disorder	0	3 (5)	3 (5)
Hemorrhage intracranial	0	2 (3)	2 (3)
Face edema	0	1(2)	1 (2)
Hypokalemia	0	1 (2)	1 (2)
Platelet count decreased	0	1 (2)	1 (2)
Pleural effusion	0	1 (2)	1 (2)
Pneumonia	0	1 (2)	1 (2)
Patients with at least one AESI	0	17 (29)	17 (26)
Cognitive effects	0	16 (27)	16 (25)
Memory impairment	0	12 (20)	12 (19)
Cognitive disorder	0	4 (7)	4 (6)
Confusional state	0	0	0
Encephalopathy	0	0	0
Intracranial bleeding	0	2 (3)	2 (3)
Intracranial hemorrhage	0	2 (3)	2 (3)
Cerebral hemorrhage	0	0	0
Subdural hematoma	0	0	0

Abbreviations: AESI, adverse event of special interest; TRAE, treatment-related adverse event.

AESIs are shown by category and then by the Medical Dictionary for Regulatory Activities Preferred Term.

Overall, 17/65 (26%) patients experienced at least one AE of special interest (AESI), all of which occurred in the 300-mg dose group (Table 4). Of these, 16 (25%) patients had an AESI classified as a cognitive effect and 2 (3%) patients experienced an AESI classified as intracranial bleeding.

**Table:** 

Primary Assessment Method		
Title	Efficacy of avapritinib in the PDGFRA D842V population (cohort 1)	Efficacy of avapritinib in the fourth- or later-line GIST population (csohort 2)
Number of patients evaluated for efficacy	28	23
Notes	73 patients were screened and 65 enrolled in the phase I/II study	

**Table:** 

Evaluation Method	IRRC-assessed ORR per mRECIST v1.1		IRRC-assessed ORR per mRECIST v1.1	
Response assessment	*N*	%	*N*	%
CR	0	0	0	0
PR	21	75	5	22
SD	5	18	13	57
PD	1	4	3	13

## Outcome Notes: Efficacy

### Efficacy of Avapritinib in the PDGFRA D842V Population in Cohort 1

The efficacy population included patients who received a starting dose of 300 mg, including 28 patients in the PDGFRA D842V population (3 in phase I and 25 in phase II). At the data cutoff date, the median follow-up was 8.6 months in the PDGFRA D842V population.

**Figure 1.**
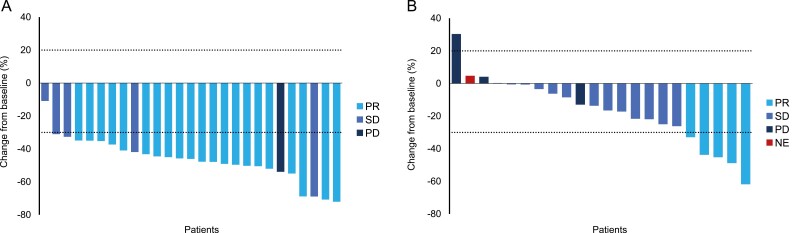
IRRC-assessed clinical outcomes. (**A**) Best percentage change from baseline in the sum of longest diameters in PDGFRA D842V-mutant gastrointestinal stromal tumors. (**B**) Best percentage change from baseline in the sum of longest diameters in patients who received avapritinib as fourth- or later-line therapy. Abbreviations: IRRC, Independent Radiology Review Committee; NE, not evaluable; PD, progressive disease; PDGFRA, platelet-derived growth factor receptor alpha; PR, partial response; SD, stable disease.

**Figure 2.**
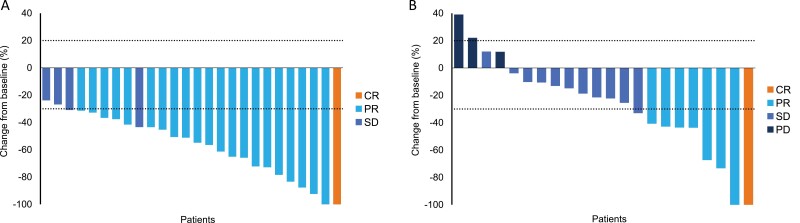
Investigator-assessed clinical outcomes. (**A**) Best percentage change from baseline in the sum of longest diameters in patients with PDGFRA D842V-mutant gastrointestinal stromal tumors and (**B**) percentage change from baseline in the sum of longest diameters in patients who received avapritinib 300 mg as fourth- or later-line therapy.

Among 28 patients with PDGFRA D842V-mutant GIST receiving a starting dose of 300 mg, partial response (PR) was achieved in 21 patients as measured by IRRC assessment, resulting in an ORR of 75% (95% CI: 55%–89%) (Table 5). The CBR was 86%. By the investigator assessments, one patient achieved complete response (CR) (4%; 95% CI: 0%–18%) and 21 achieved PR (75%; 95% CI: 55%–89%), with an ORR of 79% (95% CI: 59%–92%) and CBR of 86% (Table 6). The best percentage changes of the patient’s target lesion from baseline in the PDGFRA D842V population, as assessed by the IRRC and investigator, are shown in Figs. 1A and 2A. Five patients had PFS events, including one death. The median PFS was not reached per the IRRC assessment.

**Table 5.** Independent Radiology Review Committee-assessed anti-tumor efficacy

Response	*PDGFRA* D842V (*n* = 28) *n* (%) [95% CI]	4L+ (*n* = 23) *n* (%) [95% CI]
Objective response rate^a^	21 (75) [55-89]	5 (22) [8-44]
Best overall response		
Complete response	0	0
Partial response	21 (75) [55-89]	5 (22) [8-44]
Stable disease	5 (18) [6-37]	13 (57) [35-77]
Progressive disease	1 (4) [0-18]	3 (13) [3-34]
Not available	1 (4)	1 (4)
Not evaluable	0	1 (4)
Clinical benefit rate^b^	24 (86) [67-96]	13 (57) [35-77]

^a^Includes partial and complete responses.

^b^Complete responses + partial responses + stable disease (maintained for at least 4 cycles after treatment initiation).

Abbreviations: 4L+, patients who received avapritinib as fourth- or later-line therapy; CI, confidence interval; *PDGFRA*, platelet-derived growth factor receptor alpha.

**Table 6.** Investigator-assessed anti-tumor efficacy (patients with *PDGFRA* D842V-mutant gastrointestinal stromal tumors and patients who received avapritinib 300 mg as fourth- or later-line therapy)

	*PDGFRA* D842V (*n* = 28) *n* (%) [95% CI]	4L+ (*n* = 23) *n* (%) [95% CI]
Objective response rate^a^	22 (79) [59-92]	8 (35) [16-57]
Best overall response		
Complete response	1 (4) [0-18]	1 (4) [0-22]
Partial response	21 (75) [55-89]	7 (30) [13-53]
Stable disease	5 (18) [6-37]	11 (48) [27-69]
Progressive disease	0	3 (13) [3-34]
Not available	1 (4)	1 (4)
Clinical benefit rate^b^	24 (86) [67-96]	13 (57) [35-77]

^a^Includes partial and complete responses.

^b^Includes complete responses, partial responses and stable disease maintained for at least 4 cycles after treatment initiation (*n* = 2 in *PDGFRA* D842V and *n* = 4 in 4L+).

Abbreviations: 4L+, patients who received avapritinib as fourth- or later-line therapy; CI, confidence interval; *PDGFRA*, platelet-derived growth factor receptor alpha.

**Figure 3.**
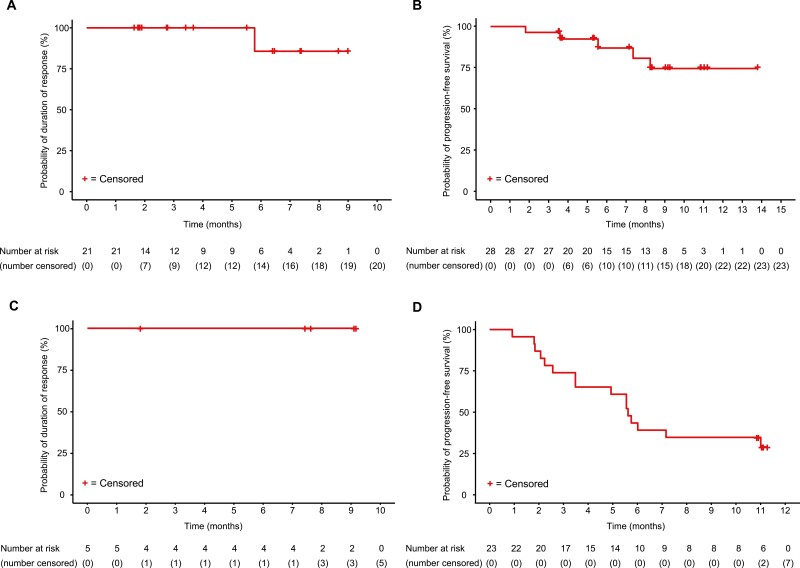
Kaplan-Meier plot of IRRC-assessments. (**A**) Duration of response of the PDGFRA D842V population, (**B**) progression-free survival of the PDGFRA D842V population, (**C**) duration of response of the 4L+ population, and (**D**) progression-free survival of the 4L+ population. Response was assessed by the IRRC per the modified Response Evaluation Criteria in Solid Tumors v1.1. Abbreviations: 4L+, patients who received avapritinib as fourth- or later-line therapy; IRRC, Independent Radiology Review Committee; PDGFRA, platelet-derived growth factor receptor alpha.

**Figure 4.**
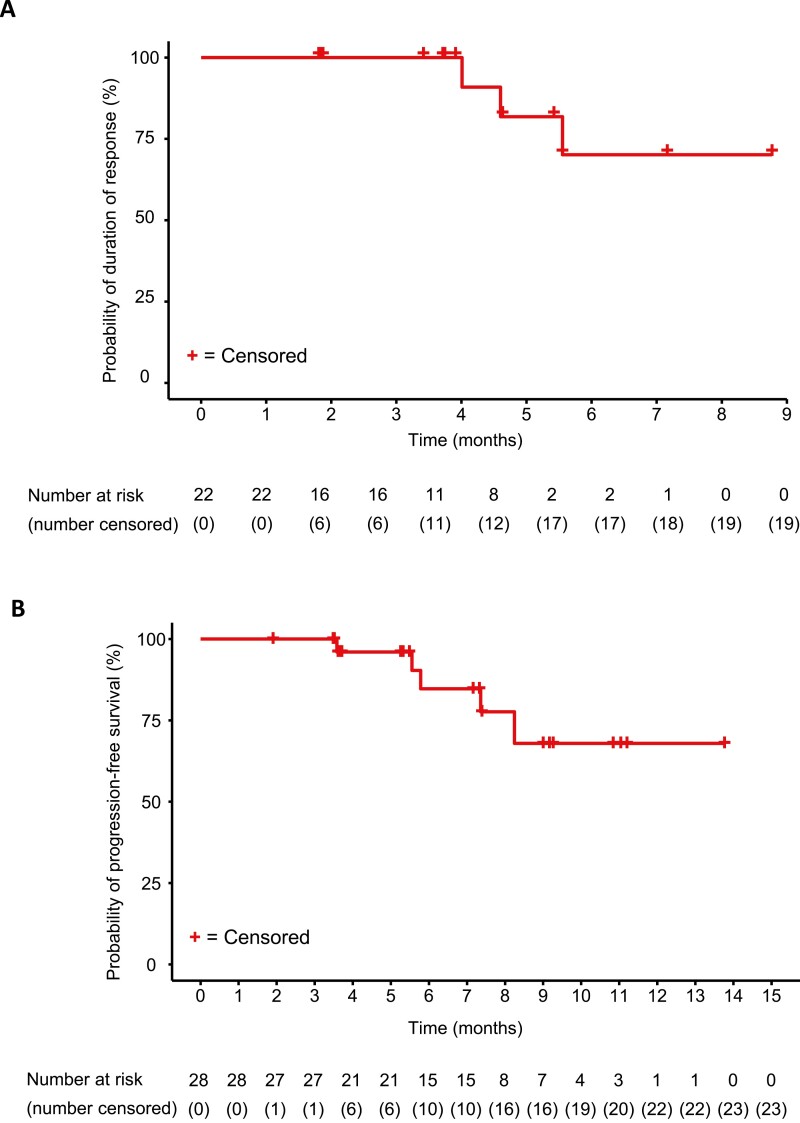
Kaplan-Meier plots of investigator assessments. (**A**) Duration of response and (**B**) progression-free survival in patients with PDGFRA D842V-mutant gastrointestinal stromal tumors.

As assessed by the IRRC, Kaplan-Meier plots of the DOR and PFS indicated that the mean DOR and median PFS were not reached in the PDGFRA D842V population receiving the 300-mg dose (Fig. 3, A and B). The 12-month OS rate was 92% (95% CI: 72%–98%). Kaplan-Meier plots of the DOR and PFS as assessed by the investigator are shown in Fig. 4, A and B.

Anti-tumor efficacy assessed by Choi criteria in the PDGFRA D842V population showed that 25/28 patients achieved PR and the ORR was 89% (95% CI: 72%–98%). The CBR per Choi criteria was 89% (95% CI: 72%–98%) (Table 7). OS data are currently immature and cannot be calculated.

**Table 7.** Choi-assessed anti-tumor efficacy (patients with *PDGFRA* D842V-mutant gastrointestinal stromal tumors and patients who received avapritinib 300 mg as fourth- or later-line therapy)

	PDGFRA D842V (*n* = 28) *n* (%) [95% CI]	4L+ (*n* = 23) *n* (%) [95% CI]
Objective response rate^a^	25 (89) [72-98]	12 (52) [31-73]
Best overall response		
Complete response	0	0
Partial response	25 (89) [72-98]	12 (52) [31-73]
Stable disease	2 (7) [1-24]	6 (26) [10-48]
Progressive disease	0	4 (17) [5-39]
Not available	1 (4)	1 (4)
Clinical benefit rate^b^	25 (89) [72-98]	13 (57) [35-77]

^a^Includes partial and complete responses.

^b^Complete responses, partial responses, and stable disease (maintained for at least 4 cycles after treatment initiation).

Abbreviations: 4L+, patients who received avapritinib as fourth- or later-line therapy; CI, confidence interval; *PDGFRA*, platelet-derived growth factor receptor alpha.

### Efficacy of Avapritinib in the Fourth- or Later-line GIST Population in Cohort 2

The efficacy population in cohort 2 consisted of 23 patients who received at least 3 previous lines of TKI therapy (ie, in the fourth- or later-line setting) and were without PDGFRA D842V mutation. These patients received a starting dose of 300 mg. At the data cutoff date, the median follow-up was 11.8 months in the fourth- or later-line treatment group.

Per IRRC assessment, PR was achieved in 5 patients (ORR 22%; 95% CI: 8%–44%) (Table 5). Per investigator assessment, one achieved CR and 7 achieved PR (ORR 35%; 95% CI: 16%–57%) (Table 6). The CBR for patients in the fourth- or later-line setting, as assessed by both investigators and the IRRC, was 57% (95% CI: 35%–77%). The best percentage changes in the patients’ target lesions from baseline, assessed by the IRRC and investigator, are shown in Figs. 1B and 2B.

**Figure 5.**
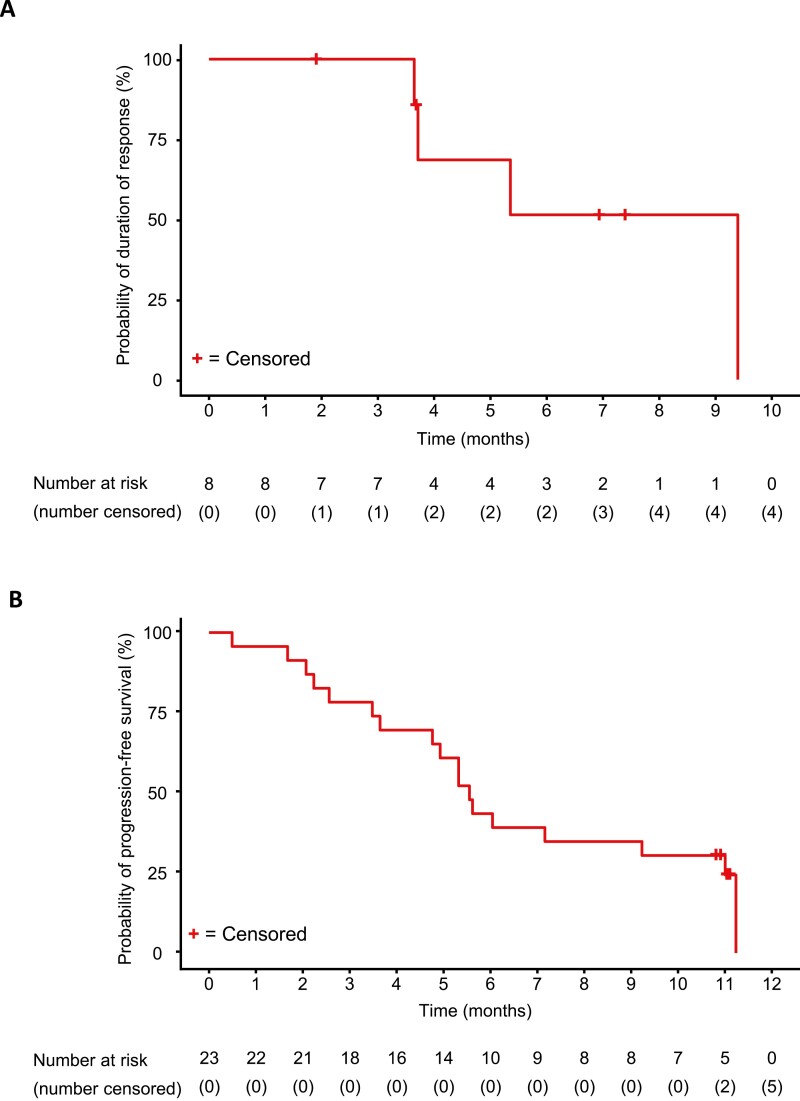
Kaplan-Meier plots of investigator assessments. (**A**) Duration of response and (**B**) progression-free survival of patients who received avapritinib 300 mg as fourth- or later-line therapy.

Kaplan-Meier plots of the DOR and PFS indicated that the IRRC-assessed median DOR in the fourth- or later-line treatment population was not reached and the median PFS was 5.6 months (95% CI: 3.5-11.0) (Fig. 3, C and D). The median investigator-assessed DOR was 9.4 months (95% CI: 3.7–not evaluable) and the PFS was 5.6 months (95% CI: 3.7-9.2) (Fig. 5, A and B). The 12-month OS rate was 61% (95% CI: 38%–77%).

Twelve patients achieved PR with an ORR assessed by Choi criteria of 52% (95% CI: 31%–73%), and the CBR per Choi criteria was 57% (95% CI: 35%–77%) (Table 7). OS data are currently immature and cannot be calculated.

## Assessment, Analysis, and Discussion

**Table:** 

Completion	Study completed
Investigator’s assessment	Active and should be pursued further

The present phase I/II bridging study showed that avapritinib had a generally tolerable safety profile in patients with advanced GIST in China. Most treatment-emergent adverse events (TEAEs) were grade 1 or 2 and were manageable. In the 300-mg (RP2D) dose group, 25% of patients experienced serious TRAEs. The most frequent TRAEs included anemia (80%) and blood bilirubin increased (75%). Based on clinical observation, treatment could have contributed to a further decrease in hemoglobin for patients with anemia at baseline. Concerning another common AE, bilirubin increase, we found that this AE was primarily associated with indirect bilirubin increase, but not direct bilirubin increase. Additionally, symptomatic liver injury was not observed simultaneously in these patients. Similarly, although creatine kinase increase was also a common AE in this study, none of the events resulted in significant clinical symptoms.

The AESIs observed in the study are consistent with the previously reported safety profiles for avapritinib. Cognitive effects related to avapritinib (memory impairment, cognitive disorder, confusional state, and encephalopathy) seem to be reversible and can be managed with dose reductions or brief interruptions. Recognition and awareness of these AEs are important for patient management.^7^ The mechanism of action of cognitive effects is still unclear and should be further investigated.

In the NAVIGATOR study, avapritinib demonstrated unprecedented, durable anti-tumor activity in heavily pretreated patients with advanced GIST harboring PDGFRA D842V mutations, providing an ORR of 91% (51/56 patients).^7^ In our study, which included a PDGFRA D842V patient population, the IRRC-assessed ORR was 75% (21/28 patients), and the CBR was 86%. Investigator-assessed ORR was 79% (22/28 patients), consistent with the IRRC assessment. Given that other therapeutic agents for GIST do not target D842V-mutant PDGFRA,^8-10^ and based on the above efficacy, avapritinib may be a potential option for addressing the well-recognized unmet need in this Chinese patient population.

The NAVIGATOR study assessed efficacy and safety in patients without PDGFRA D842V mutation in the fourth- or later-line cohort and reported an ORR of 17% and median PFS of 3.7 months.^11^ In this study, the IRRC-assessed ORR in patients in the fourth- or later-line setting was 22%, and the median PFS was 5.6 months, which is consistent with the investigator-assessed median PFS of 5.6 months. Of note, the median PFS with avapritinib (5.6 months) in patients in the fourth- or later-line setting was similar to data reported for ripretinib (5.5 months in a phase I study^12^ and 6.3 months in a phase III study^8^). However, the ORR for avapritinib as fourth- or later-line monotherapy (22% [5/23 patients]) is competitive in comparison with the ORR reported for either ripretinib (7.2% [6/83 patients] in patients in the fourth-line setting in the phase I study,^12^ 9.4% [8/85 patients] in patients in the fourth- or later-line setting in the phase III study^8^), or regorafenib (4.5% [6/133 patients] in patients in the third- or later-line setting in the phase III GRID study^13^). The notable response rates observed in the PDGFRA D842V and fourth- or later-line treatment populations suggest that avapritinib may contribute to downstaging GIST for possible resection. TKIs are the cornerstone of treatment for advanced GIST, but limited treatment options are available for patients with GIST who have received at least 3 lines of previous treatment.^14^ The ORR (22%) with avapritinib in patients in the fourth- or later-line cohort in this analysis is numerically higher than the ORRs of approved therapies for unresectable GIST.^15^ The tolerable safety profile of avapritinib suggests that it could be a new fourth- or later-line monotherapy option for GIST management.^16,17^

In this China-specific study, we conducted an exploratory efficacy analysis per IRRC assessment according to Choi criteria. ORRs were 89% and 52% in the PDGFRA D842V and fourth- or later-line populations, respectively, indicating visible differences between the results evaluated per Choi and mRECIST criteria and a numerically higher ORR per Choi criteria. The common metastatic sites of GIST are the peritoneum and liver,^18^ and lesions are multiple and highly heterogeneous. To some extent, the selection of target lesions per mRECIST may limit the comprehensiveness and accuracy of efficacy evaluations. Therefore, in clinical practice, the concept of Choi criteria is widely accepted and applied, sometimes in preference to mRECIST, to evaluate GIST and provide another meaningful reference for clinical diagnosis, leading to optimal treatment regimen selection.

This study had limitations, including the open-label design, lack of a control/comparator group, and the small sample size, because of the low rate of incidence and mutation. The key strength of this study was that it targeted Chinese patients with PDGFRA D842V-mutant GIST, and those who had received at least 3 prior lines of therapy, which represents a currently important unmet need.

In conclusion, avapritinib provided pronounced anti-tumor activity in patients with PDGFRA D842V mutations and those who had received at least 3 prior lines of therapy. The demonstrated therapeutic activity and favorable safety profile of avapritinib in the PDGFRA D842V study population indicate that avapritinib may be an effective treatment for GIST patients with PDGFRA D842V mutations. The National Medical Products Administration has approved avapritinib as SOC for patients with PDGFRA D842V-mutant GIST in China based on this study, in combination with the global NAVIGATOR study.

## Data Availability

The datasets generated and analyzed during the current study are not publicly available due to the internal policies of CStone Pharmaceuticals.

## Appendix: Supplemental Methods

### Study Design

This study was initiated in August 2019 and registered with ClinicalTrials.gov; identifier: NCT04254939. The clinical study protocol and related documentation were approved by an Independent Ethics Committee. All procedures performed in this study followed the International Council for Harmonisation Good Clinical Practice Guidelines, the Declaration of Helsinki, and other relevant regulatory requirements.

### Eligibility Criteria

For phase I, enrollment criteria were surgically unresectable or metastatic gastrointestinal stromal tumor (GIST) confirmed by histology or cytology, and either progression after treatment with imatinib and at least one other tyrosine kinase inhibitor (TKI) or intolerance to standard of care (SOC) or lack of available SOC, or presence of a mutation in the platelet-derived growth factor receptor alpha (*PDGFRA*) gene causing the D842V substitution in the PDGFRA protein.

Patients were excluded from the study if, within 14 days prior to the first administration of the study drug, they had received antineoplastic drugs, received neutrophil growth factor support, received a potent inhibitor of cytochrome P450 3A4 or a potent or intermediate induction agent, or undergone surgery. Exclusion criteria also included impaired liver function tests, impaired renal function based on creatinine clearance, low platelet or neutrophil counts, and anemia (hemoglobin <9 g/dL).

### Study Procedures

All study observations were conducted at outpatient visits unless hospitalization was required for any reason. After providing informed consent, a screening period began within 28 days (4 weeks) before the first avapritinib dose. Safety monitoring was performed at the study center, and the tumor status of patients was evaluated by computed tomography (CT) or magnetic resonance imaging (MRI). Chest CT, and CT or MRI of the abdomen and pelvis, were performed at screening with intravenous contrast injection. All patients received radiological examination of the bodily areas containing the target and non-target lesions on day 1 of treatment cycle 3, and every two cycles thereafter. All patients in phase I and 2 participated in the end of treatment visit 14 (± 7) days after the last dose of the study drug. A safety follow-up was conducted by telephone 30 (± 7) days after the last dose of the study drug, or when the patient started another anti-tumor treatment, to determine the resolution/recovery status of adverse events. Subsequent follow-up was conducted every 3 months to evaluate the disease course, subsequent anti-tumor treatment, and survival until disease progression or death (or other withdrawal, including withdrawal of consent). *PDGFRA* D842V mutations were identified at a local or sponsor-designated laboratory using archived or newly biopsied tumor tissue.

### Outcomes

Dose-limiting toxicities (DLTs) were defined as any treatment-emergent adverse events of grade ≥3 occurring during cycle 1 of the treatment phase that could not be attributed to any cause other than treatment with avapritinib.

Clinical benefit rate was defined as the proportion of patients achieving complete response, partial response, or stable disease, each maintained for at least 4 periods after beginning treatment.

### Sample Size Calculations

The sample size of the phase I part was dependent on the observed safety profile but was expected to be 9 to 12 patients. The sample size target for the phase II part was based on the calculation of the 95% confidence interval of the objective response rate with estimated sample sizes of 25 patients in cohort 1 (*PDGFRA* D842V mutation) and 30 patients in cohort 2 (3L and 4L+ patients).

### Analysis Sets

The dose-determining set included all patients in the phase I study who met the minimum exposure standard in the first dose period and completed the day 28 follow-up visit or those who stopped treatment due to a DLT. The efficacy analysis set included all patients who received at least one dose of the study drug and had a measurable baseline lesion.

The safety population included all Chinese patients (phase I patients and phase II 3L+ patients) with unresectable or metastatic GIST. The efficacy population included patients in two groups: those with a *PDGFRA* D842V mutation and those treated with at least three prior lines of therapy (i.e., 4L+).
